# Effect of *Brassica napus* cultivar on cellulosic ethanol yield

**DOI:** 10.1186/s13068-015-0278-z

**Published:** 2015-07-11

**Authors:** Ian P. Wood, Nikolaus Wellner, Adam Elliston, David R. Wilson, Ian Bancroft, Keith W. Waldron

**Affiliations:** The Biorefinery Centre, Institute of Food Research, Norwich Research Park, Colney, Norwich NR4 7UA UK; Analytical Sciences Unit, Institute of Food Research, Norwich Research Park, Colney, Norwich NR4 7UA UK; Department of Biology, University of York, Heslington, York YO10 5DD UK

**Keywords:** Bioethanol, Biomass saccharification, Crop cultivars, Cultivar variation, Dicot, Dicotyledonous, Oilseed rape, Fermentation, Pretreatment, Rapeseed straw

## Abstract

**Background:**

Intraspecific variations in biomass composition are likely to influence their suitability for biorefining. This may be particularly important in species such as *Brassica napus*, which contain many different crop types bred for different purposes. Here, straw derived from 17 *B. napus* cultivars, of varying crop types, were steam exploded, saccharified and fermented to establish differences in biomass composition relevant to cellulosic ethanol production.

**Results:**

Despite being grown and processed in the same manner, straw from the various cultivars produced different saccharification and fermentation yields after processing. Fermentation inhibitor abundances released by steam explosion also varied between genotypes. Cultivars with glucan-rich straw did not necessarily produce higher saccharification or ethanol yields after processing. Instead, the compositions of non-cellulosic components were more reliable indicators of substrate quality. The abundance of pectins and arabinogalactans had the greatest influence on saccharification efficiency between straw genotypes.

**Conclusions:**

In dicotyledonous species, such as *B. napus*, variations in the abundance of pectins between crop cultivars are likely to influence processing efficiency for bioethanol production. Knowledge of these genotypic variants provides targets for plant breeding and could aid in the development of improved cellulase cocktails.

## Background

Variations in biomass composition are likely to influence their suitability for exploitation. Therefore, if biomass is to be used to create sustainable products, such as ethanol, we must first understand the compositional variants that determine substrate quality [1]. If the chemical basis of biomass usability can be identified, both feedstock and processing conditions can be improved.

Substrate variation is an important consideration for industry for many reasons. If sufficient variation exists between cultivars, it could be exploited by crop breeders to improve feedstock quality [2]. On the other hand, cultivar variation may be undesirable to biorefinery operators who are likely to require uniform and predictable yields regardless of the biomass source.

As highlighted by other researchers, biomass composition can vary considerably [3], even between members of the same species with similar plant architectures [2, 4], such as wheat [2, 4–6], rice [7] and maize [8, 9]. Intraspecific variations in these monocotyledonous biomass sources are likely to be determined by the abundance of plant tissue types [4, 10], which vary depending on agronomic conditions and genotype.

If commodity chemicals are to be produced from biomass, agricultural residues from dicotyledonous plants, such as *Brassica napus* straw, may also be used [11, 12]. These species have very different cell wall structures to monocot plants [13]. Unlike many crop species, *B. napus* has been bred to produce a range of products from vegetable oil (oilseed rape (OSR)) to animal fodder (fodder rape). Consequently, considerable phenotypic and genotypic variation exists within *Brassica* species [14]. It is therefore likely that these genetic and phenotypic differences will also influence lignocellulose composition through differences in cell wall (CW) chemistry and tissue abundances. In a biorefinery context, where (ligno)cellulose is converted to monomeric sugars and fermented to produce chemicals and fuels [1, 11], it is likely that these variations will influence process efficiency and yields.

Fermentable sugars can be released from lignocellulose in a number of ways. However, one of the most promising production routes currently available involves pretreatment by steam explosion, followed by enzymatic hydrolysis [15]. Steam explosion modifies the chemical composition [16] and polymeric structure of *B. napus* straw [17]. The resulting material is therefore more amenable to enzymatic saccharification [18]. Previous studies have shown that steam explosion improves methane yields during anaerobic digestion [16] and fermentable sugar yields after enzymatic saccharification [17, 18]. These studies revealed that retention of uronic acid- and xylose-containing compounds were the important process-specific factors limiting initial hydrolysis rate and overall reducing sugar yield, respectively [18]. It would be interesting to see if intraspecific variations in these components were also important determinants of substrate quality.

Although process-dependent differences have been explored using *B. napus* straw from a single genotype [16–18], little is known about the effect that variations in straw composition have on saccharification yields with this feedstock. Furthermore, although significant differences in saccharification yields are known to exist within members of the same species, the precise chemical basis for these variations is not fully understood.

Therefore, this work aimed not only to determine differences in straw quality between cultivars using pilot-scale processing but also to relate those differences to straw composition. To do this, straw derived from a selection of OSR cultivars and other crop types of the same species (*B. napus*) was pretreated at near-optimal conditions [9] using pilot-scale steam explosion. The chemical composition of the original material, pretreated substrates and products released during processing were established. IR spectra were also taken from these materials which gave an insight into their polymeric structure. Monomeric glucose (Glc) and ethanol yields were quantified after hydrolysis and simultaneous saccharification and fermentation (SSF), respectively. This data allowed differences in product yields between cultivars to be related to differences in straw composition.

## Results and discussion

### The carbohydrate composition of *B. napus* straw differed between genotypes

Despite being grown, harvested, stored and analysed under the same conditions, significant variations in the abundance of constituent sugars were observed between *B. napus* straw from different cultivars (Table 1). The mean moisture content of the straw was ca. 9.5 % and did not differ significantly between cultivars (Table 1).

**Table 1:** Sugar composition of untreated *B. napus* straw derived from different cultivars

Cultivar name	Composition (g/kg original air-dry straw)									
	Glc	Xyl	UA	Man	Gal	Ara	Rha	Fuc	MC	Other
Canard	315	139	44	16	11	11	4	1	89	370
Canberra x Courage	319	146	39	16	12	9	5	1	95	359
Darmor	299	133	40	18	12	10	5	2	100	381
Erglu	339	145	37	20	14	12	5	2	100	325
Hansen x Gaspard	353	130	38	19	14	10	5	2	100	330
Judzae	342	149	40	21	15	11	6	3	99	314
Licrown x Express	377	146	38	24	13	11	6	1	96	288
Madrical x Recital	331	137	44	20	13	13	5	2	101	334
Major	315	130	44	19	12	9	5	1	95	369
POH285, Bolko	334	145	47	18	13	11	5	1	84	342
Quinta	289	125	34	15	14	14	5	1	95	408
Ramses	292	115	40	15	13	13	5	1	86	419
Sensation NZ	349	148	34	19	13	9	5	1	85	337
Shannon x Winner	320	140	45	18	13	12	5	1	107	339
Slapka Slapy S3	310	134	33	17	11	10	5	1	101	379
Slovenska Krajova	321	135	44	16	12	10	5	1	86	370
York	349	150	40	20	13	9	5	1	102	312
Mean	327	138	40	18	13	11	5	1	95	352
Range	88	35	14	9	4	5	2	2	23	131
Range (% mean)	27	26	35	50	31	48	35	113	24	37
ANOVA (*p* value)	<0.05	<0.01	<0.001	<0.001	<0.05	<0.001	0.564	<0.01	0.399	–

Values were calculated with a relative standard deviation (RSD) of 3.9, 3.2, 7.3, 2.6, 4.8, 4.8, 6.8, 12.1 and 8.1 % for Glc, Xyl, UA, Man, Gal, Ara, Rha, Fuc and MC, respectively

*Glc* glucose, *Xyl* xylose, *UA* uronic acids, *Man* mannose, *Gal* galactose, *Ara* arabinose, *Rha* rhamnose, *Fuc* fucose, *MC* moisture content, *Other* other non-carbohydrate matter by difference, *ANOVA* one-way analysis of variance

### After pretreatment, the compositions of the water-insoluble residues were more uniform but still varied between cultivars

Straw derived from each cultivar (1 kg) was steam exploded into hot water at near-optimal conditions (210 °C, 10 min). The sugar compositions of the steam-exploded water-insoluble solids were then established to see if genotypic variation in composition observed between the untreated straw of different cultivars was retained after pretreatment (Table 2).

**Table 2:** Matter recoveries and monomeric sugar composition of straw steam exploded at 210 °C, 10 min derived from different cultivars

Cultivar name	Recovery (g/kg original FW)		Composition (g/kg pretreated material DW)								
	Mass DW	MC (%)	Glc	Xyl	UA	Man	Gal	Ara	Rha	Fuc	Other
Canard	340	65	416	30	13	2	1	5	2	Trace	544
Canberra x Courage	514	67	413	30	13	2	2	5	2	Trace	546
Darmor	447	68	426	30	14	2	1	4	2	Trace	536
Erglu	488	66	394	30	19	2	2	5	2	Trace	566
Hansen x Gaspard	520	61	430	33	11	Trace	2	4	2	Trace	528
Judzae	554	65	400	34	17	Trace	2	4	2	Trace	558
Licrown x Express	528	71	407	27	12	2	1	3	2	Trace	558
Madrical x Recital	616	65	427	29	13	Trace	1	3	2	Trace	539
Major	460	71	427	26	13	Trace	1	0	2	Trace	543
POH285, Bolko	447	71	437	32	19	Trace	1	1	2	Trace	528
Quinta	513	69	361	31	13	Trace	1	4	1	Trace	602
Ramses	478	61	439	28	17	Trace	1	2	2	Trace	528
Sensation NZ	542	64	384	31	12	2	2	3	2	Trace	576
Shannon x Winner	523	66	395	26	16	Trace	1	1	2	Trace	576
Slapka Slapy S3	480	70	413	26	12	1	1	3	2	Trace	555
Slovenska Krajova	475	71	377	32	12	3	1	5	2	Trace	581
York	503	65	455	28	17	Trace	0	0	2	Trace	515
Mean	496	67	412	30	14	–	1	3	2	–	552
Range	276	10	94	8	8	–	2	5	1	–	87
Range (% mean)	56	15	23	27	56	–	135	171	47	–	16
ANOVA (*p* value)	–	<0.01	<0.001	<0.001	0.511	–	0.287	<0.05	<0.05	–	–

Values were calculated with a RSD of 2.5, 3.0, 3.5, 16.7, 78.1, 39.1, 67.3 and 7.5 % for MC, Glc, Xyl, UA, Man, Gal, Ara and Rha, respectively

*Glc* glucose, *Xyl* xylose, *Ara* arabinose, *Gal* galactose, *Fuc* fucose, *UA* uronic acids, *Other* other non-carbohydrate matter, *MC* moisture content, *FW* fresh weight, *DW* dry weight

The yields of washed, steam-exploded material on a dry-weight basis as a function of the original material are shown in Table 2. These show that approximately half of the dry matter was lost from the biomass during the pretreatment. The most likely explanation for this is the breakdown and solubilisation of non-cellulosic polysaccharides and other low-molecular-weight substances, as well as the loss of small particulate matter during the cyclone and washing stages (e.g. [19]).

Nevertheless, biomasses from different cultivars were treated identically and processed in a random order in relationship to their CW compositions. Therefore, the relative differences in chemistry of the pretreated material and saccharification yields are likely to reflect the genotypic differences in biomass composition. After steam explosion, mannose (Man), galactose (Gal) and fucose (Fuc) were almost completely removed from the water insoluble fraction (<5 % of the original remained) (Table 2). Likewise, other non-cellulosic sugars (xylose (Xyl), uronic acids (UA), arabinose (Ara) and rhamnose (Rha)) were also removed but retained a higher proportion of their sugars in the pretreated residue (10–20 % of the original). By contrast, up to 80 % of the original Glc present in the original material was retained in the steam-exploded residue.

After steam explosion, the largest quantitative difference between substrates produced from different cultivars was in the abundance of glucan retained in the water-insoluble material (Table 2). The glucan content broadly correlated with that of the original straw. Although present in much smaller quantities, larger proportional variations were observed in the reduced retention of non-cellulosic carbohydrates containing Xyl, Ara and Rha between cultivars. Straw from particular cultivars, such as Canard, retained small quantities of arabinan after steam explosion (≈5 g/kg), as others, such as York, retained almost none (Table 2). These results indicated considerable varietal differences in the pretreatment lability of non-cellulosic polysaccharides.

### Polymeric differences in biomass composition between cultivars revealed using Fourier transform infrared spectroscopy

**Fig. 1:**
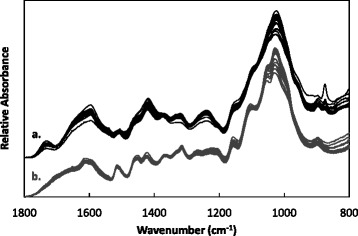
Average FT-IR spectra collected from straw, derived from different cultivars before (**a**) and after (**b**) steam explosion at 210 °C, 10 min

Fourier transform infrared (FT-IR) spectroscopy has been used extensively to probe the structure of plant CWs [20, 21]. Here, spectra for OSR straw from different cultivars before and after steam explosion were used to assess cultivar-specific differences at a polymeric level (Fig. 1).

Spectra collected from untreated straw showed greater variation between cultivars than those from the same materials after SE. The largest spectral differences were observed at wavenumbers typically associated with structural carbohydrates—cellulose, hemicellulose and pectic structures—875, 1020, 1240, 1315, 1420, 1600 and 1730 cm^−1^ [21]. Particular cultivars showed above-average deviation in absorbance at certain wavelengths. For example, Ramses straw exhibited higher absorbance at 875 cm^−1^ (C1–H bending in xyloglucan (XG) and cellulose) compared to other cultivars. Similarly, Hansen x Gaspard showed above-average absorbance at 1600 cm^−1^ (COO^−^ asymmetric stretching), suggesting differences in pectic cross-linking [21].

After pretreatment, spectra taken from the residues of different cultivars were more uniform (Fig. 1). The largest variation between cultivars was observed at wavenumbers related to non-cellulosic polysaccharide abundances: 1020 cm^−1^ (C–O stretching, C–C stretching in XG and pectins) and 1155 cm^−1^ (C–O–C glycosidic linkages in xylan) [20]. Spectral variations between cultivars identified at other wavenumbers were diminished following steam explosion, reflecting the extent of component removal from the biomass.

### Variation in fermentation inhibitor release differed between cultivars

We previously showed that significant quantities of organic breakdown compounds are produced from *B. napus* straw when steam exploded at severities required to achieve reasonable saccharification yields (>60 %) [18]. Many of these have the capacity to inhibit downstream processes—particularly fermentation [22]. In the current study, the abundance of four common inhibitory compounds (furfural, hydroxymethylfurfural, acetic acid and formic acid) released into the pretreatment liquor varied significantly between cultivars (*p* < 0.001) (Table 3). This variation in fermentation inhibitor production could be exploited to limit the production of compounds that are detrimental to downstream processes.

**Table 3:** Concentration of organic acids and furfural derivatives retained in the pretreatment liquors of straw derived from different cultivars

Cultivar name	Volume (L)	Concentration (g/L pretreated liquor)			
		Acetic	Formic	2FA	HMF
Canard	6.60	2.86	2.02	0.51	0.15
Canberra x Courage	7.08	2.68	2.04	0.54	0.16
Darmor	7.13	2.75	2.11	0.46	0.11
Erglu	6.95	2.72	1.97	0.32	0.15
Hansen x Gaspard	6.96	2.35	1.77	0.49	0.12
Judzae	7.00	2.25	1.81	0.37	0.11
Licrown x Express	6.25	3.20	2.08	0.50	0.18
Madrical x Recital	6.50	2.76	2.02	0.42	0.13
Major	6.84	2.59	1.91	0.34	0.14
POH285, Bolko	7.00	3.13	2.18	0.46	0.18
Quinta	6.76	2.91	1.95	0.67	0.20
Ramses	6.55	2.68	1.60	0.45	0.23
Sensation NZ	7.15	2.43	1.96	0.28	0.11
Shannon x Winner	6.85	3.40	1.92	0.39	0.26
Slapka Slapy S3	6.92	3.08	1.97	0.41	0.20
Slovenska Krajova	6.78	2.39	1.50	0.76	0.17
York	6.18	3.23	2.08	0.43	0.23
Mean	6.79	2.79	1.93	0.46	0.17
Range	0.97	1.15	0.68	0.48	0.15
Range (% mean)	14	41	35	105	90

The abundance of all compounds in the hydrolysis liquors differed significantly between cultivars (ANOVA, *p* < 0.001). Values were calculated with a RSD of 1.9, 1.7, 1.9 and 5.4 % for acetic acid, formic acid, 2FA and HMF, respectively

*Acetic* acetic acid, *Formic* formic acid, *2FA* 2-furfuraldehyde, *HMF* hydroxymethylfurfural

### Straw from different cultivars obtained different hydrolysis and fermentation yields

A portion of the steam exploded biomass derived from each cultivar was converted to either Glc or ethanol by enzymatic hydrolysis or SSF, respectively, using a near-optimum cellulase dose determined previously (36 FPU/g substrate [18]) (Table 4). Although all 17 cultivars were grown, processed and analysed in the same manner, significant differences (*p* < 0.001) in product yields were observed between cultivars (Table 4).

**Table 4:** Estimated mass of reducing sugars (by DNS), glucose and ethanol produced from pretreated straw derived from different cultivars (5 % substrate, 37 FPU/g, 96 h) incubated at 50 or 40 °C, respectively

Cultivar name	Product yield (g/kg PT material)		
	DNS^a^	Glucose	Ethanol
Canard	445 ± 39	289 ± 13	142 ± 1
Canberra x Courage	510 ± 42	352 ± 23	*107* ± *19*
Darmor	483 ± 15	367 ± 22	173 ± 35
Erglu	415 ± 48	286 ± 37	154 ± 14
Hansen x Gaspard	564 ± 94	361 ± 19	*107* ± *13*
Judzae	395 ± 29	269 ± 8	171 ± 13
Licrown x Express	438 ± 14	289 ± 6	147 ± 13
Madrical x Recital	452 ±24	302 ± 45	135 ± 10
Major	484 ± 24	344 ± 19	177 ± 9
POH285, Bolko	496 ± 22	331 ± 32	146 ± 31
Quinta	460 ± 60	312 ± 44	137 ± 20
Ramses	*325* ± 39	*215 ± 20*	*91 ± 17*
Sensation NZ	423 ± 21	277 ± 21	135 ± 36
Shannon x Winner	388 ± 29	266 ± 17	125 ± 5
Slapka Slapy S3	514 ± 52	374 ± 58	185 ± 18
Slovenska Krajova	511 ± 18	332 ± 55	157 ± 9
York	456 ± 24	344 ± 21	141 ± 7
Mean	456	312	143
Range	239	159	94
Range (% mean)	52	51	66

Significant differences in product yields were observed between cultivars (ANOVA *p* < 0.001). Italicised values are atypically low when compared to most cultivars

^a^Glucose equivalent reducing groups as assayed using DNS reagent

Here, two methods were used to quantify saccharification products. Total reducing sugars in the hydrolysates was estimated using dinitrosalicylic acid (DNS) reagent and a Glc-specific assay (GOPOD) used for accurate quantification of monomeric Glc release. Reducing sugar assays typically overpredict sugar yields as other chemicals created during pretreatment, such as furfural derivatives, also contain reducing groups but have much lower mass than Glc [23]. This is particularly apparent when severe pretreatment conditions are used. Nevertheless, reducing sugar yields assayed using the DNS reagent correlated strongly with Glc yields (*p* < 0.001, *R* = 0.920, *n* = 17), demonstrating that the main variations in sugar release between cultivars related to glucan digestibility.

Ethanol yields produced by SSF generally reflected Glc yields saccharified from the material except for Hansen x Gaspard and Canberra x Courage, which obtained good saccharification yields but performed very poorly under SSF conditions (Table 4). Typically, 95 % of the Glc hydrolysed from the pretreated material was fermented to ethanol under SSF conditions. It is therefore likely that ethanol yields produced from ‘Hansen x Gaspard’ and ‘Canberra x Courage’, which only produced ≈60 % of the expected yield based on the saccharification results alone, were not indicative of the general trend in product yields observed between cultivars (Table 4). Such cultivars might provide useful model systems for identifying mechanisms that reduce fermentation efficiency. Without these outliers, monomeric Glc and reducing sugar yields correlated strongly with ethanol yields as one might expect (*p* < 0.01, *R* = 0.755 and 0.704, respectively, *n* = 15).

### Relationship between straw composition and product yields

To understand the potential relationship between cultivar straw composition and product yields, the abundance of each component sugar present in the untreated and pretreated residues were correlated with monomeric Glc and ethanol yields after processing (Table 5). Cultivars with glucan-rich straws did not necessarily produce higher monomeric Glc (*p* = 0.957, *n* = 17) or ethanol yields after processing (*p* = 0.730, *n* = 15). These results are similar to those observed in maize, where varietal differences in ethanol yield were more closely related to glucan convertibility rather than glucan content [24].

**Table 5:** Correlations between straw composition and product yields

Component	DNS*		Glucose		Ethanol	
	(g/kg PT, *n* = 17)		(g/kg PT, *n* = 17)		(g/kg PT, *n* = 15)	
	*R*	*p*	*R*	*p*	*R*	*p*
Original straw composition (g/kg FW)						
Glc	0.104	0.691 n.s.	−0.014	0.957 n.s.	0.098	0.730 n.s.
Xyl	0.054	0.837 n.s.	0.082	0.756 n.s.	0.291	0.293 n.s.
UA	−0.034	0.896 n.s.	−0.090	0.732 n.s.	−0.126	0.655 n.s.
Ara	−0.499	0.042*	−0.559	0.019*	−0.517	0.049*
Gal	−0.441	0.077 n.s.	−0.530	0.029*	−0.353	0.196 n.s.
Rha	−0.239	0.355 n.s.	−0.256	0.321 n.s.	0.202	0.470 n.s.
Man	−0.092	0.725 n.s.	−0.073	0.780 n.s.	0.240	0.390 n.s.
Fuc	−0.210	0.418 n.s.	−0.168	0.520 n.s.	0.222	0.427 n.s.
Other	−0.045	0.865 n.s.	0.002	0.994 n.s.	−0.174	0.535 n.s.
Pretreated straw composition (g/kg DW)						
Glc	0.077	0.770 n.s.	0.199	0.443 n.s.	−0.079	0.780 n.s.
Xyl	0.216	0.405 n.s.	0.022	0.934 n.s.	0.063	0.824 n.s.
UA	−0.515	0.034*	−0.378	0.135 n.s.	−0.243	0.384 n.s.
Ara	0.207	0.425 n.s.	0.051	0.845 n.s.	0.195	0.485 n.s.
Gal	0.021	0.937 n.s.	−0.139	0.594 n.s.	0.204	0.466 n.s.
Rha	0.149	0.567 n.s.	0.099	0.706 n.s.	−0.012	0.966 n.s.
Other	−0.131	0.616 n.s.	−0.220	0.396 n.s.	0.053	0.852 n.s.

Correlation coefficients (*R*) and significance values (*p*) are presented, with significant correlations (*p* < 0.05) marked with an asterisk (*)

*FW* fresh weight, *DW* dry weight, *ns* not significant

Cultivars that contained fewer Ara-containing components in their original straw tended to produce higher Glc yields after steam explosion and hydrolysis (*p* < 0.49, *n* = 16) and ethanol after SSF (*p* < 0.05, *n* = 15). Likewise, Gal composition of the original straws also negatively correlated with Glc yields (*p* < 0.03, *n* = 17) and (not significantly, *n* = 0.2) with ethanol yields. Interestingly, a low-yielding cultivar, Ramses, contained relatively high galactan and arabinan content compared to other cultivars (Table 5). It is therefore possible that particularly low saccharification yields were produced from Ramses straw because of this difference in CW chemistry.

In contrast, comparison of Glc and ethanol yields with pretreated straw chemistry showed no such correlations with non-cellulosic neutral sugars (Table 5). The most likely reason for this is that the non cellulosic neutral sugars were almost completely removed during the pretreatment process. Nevertheless, the fact that the final yields of Glc and ethanol maintained a correlation with the original straw chemistry suggests that some physical or chemical signatures still exist in the pretreated material which has a negative impact on the digestibility and fermentation stages.

These results suggest that polymers enriched in Ara and Gal such as pectins (particularly rhamnogalacturonan I (RG-I)) or arabinogalactans (AGs) are likely to influence biomass recalcitrance between *B. napus* cultivars after pilot-scale SE. Recent evidence has shown that AGs can covalently link the hemicellulose-pectin network [25]. AGs are thermally resistant CW-associated polymers [26]; therefore, it is possible that genotypic differences in the abundance of these, or similarly Ara- and Gal-rich polymers, could have a significant impact on substrate recalcitrance after pretreatment. Similarly, pectic side chains on RG-I are comprised mainly of Ara and Gal sugars, which are likely to hinder degradation—particularly when hydrolysing dicotyledonous biomass [27]. Further work, involving the more detailed characterisation of biomass, would be needed to ascertain the effect that these carbohydrates may have on CW recalcitrance.

Interestingly, variations in UA abundance retained in the pretreated solid between cultivars correlated negatively with reducing sugar yields after pretreatment and enzymatic hydrolysis (Table 5). This observation is consistent with previous work where saccharification performance of straw steam exploded from a single *B. napus* genotype was limited by severity-dependent UA retention [18]. Together, these results suggest that variations in UA retained in the pretreated material, brought about by either changes in pretreatment severity [18] or straw composition (this work), are particularly important components influencing the saccharification of OSR straw. These results also mirror those collected from other herbaceous, dicotyledonous plants such as hemp, where galacturonic acid content correlates negatively with saccharification yield, irrespective of the pretreatment technique used [28].

### Relating genotypic variation in IR spectra with variation in ethanol yields using partial least squares regression

Partial least squares (PLS) regression is a convenient way of correlating quantitative measurements with spectral data. As mentioned previously, FT-IR spectra give an overview of the constituent bonds present in the material—thereby giving information as to its polymeric structure. This can make spectral interpretations of biological material difficult, as many infrared absorbance peaks overlap. Splitting the spectral variation into successive, principal components, using multivariate analysis, makes the data more accessible: highlighting areas of the spectra that correlate with variances in quantitative measurements.

Previously, this methodology has been used to provide information on the main polymeric changes that occur in OSR straw following steam explosion at varying pretreatment severities [17]. The crucial effects that these changes in severity had on subsequent Glc (via enzymatic hydrolysis) [17] and methane generation (after anaerobic digestion) were also identified [16].

**Fig. 2:**
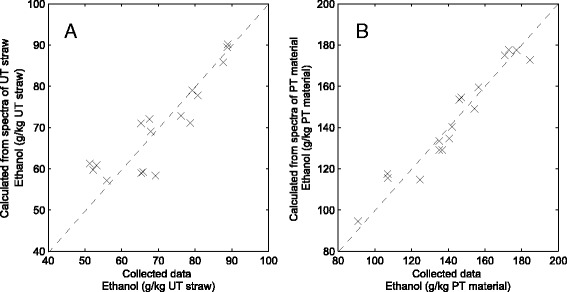
Ethanol yields predicted from FT-IR spectra compared to actual data collected from untreated (**a**) and pretreated (**b**) materials. Data predicted by fitting to six PLS components are shown

Here, PLS modelling was used to match spectral variations between cultivars to variations in ethanol yields after processing—summarising them into six PLS components (PLS 1–6). Spectra taken from the untreated straw samples were correlated with ethanol yields obtained from the same cultivar in grams per kilogram original straw (Fig. 2a). Likewise, spectra taken from pretreated cultivar straw samples were correlated with ethanol yields expressed as grams per kilogram steam-exploded straw (Fig. 2b).

**Fig. 3:**
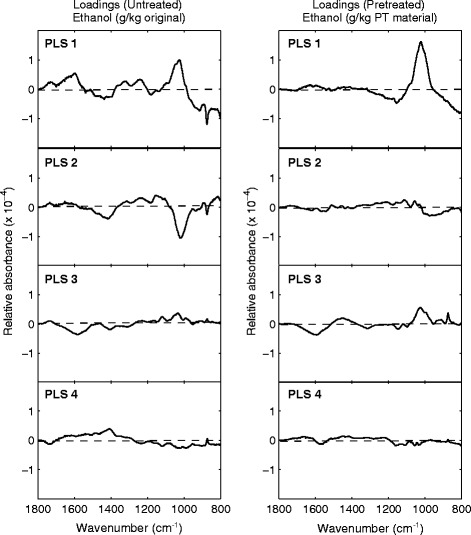
PLS loadings showing spectral variations correlated with ethanol yields in untreated (*LHS*) and pretreated (*RHS*) straw produced from different cultivars. The first four PLS components are displayed (*PLS 1–4*)

This showed that variations in FT-IR spectra collected from both untreated and pretreated straw accessions could provide reasonable estimations of the ethanol yields obtained after processing (Fig. 2). In total, the first six PLS components could describe 97–98 % of intraspecific variation in ethanol yields utilising 78–83 % of the spectral variations observed between cultivars. These models show that variations in the chemistry of the untreated and pretreated material, detected as spectral differences, can be matched to the different ethanol yields between straw accessions. The cause of these differences can be interrogated further by examining the loadings for each PLS component (Fig. 3).

The loadings for each component were therefore examined to identify what differences in polymeric associations between cultivars are likely to influence ethanol yields (Fig. 3). The majority of variation in ethanol yields observed between cultivars after steam explosion (76.83 %) could be explained by a single PLS component (PLS 1)—utilising 29.6 % of the variation in spectra collected from the pretreated residues (Fig. 3, RHS). This spectral variation was mostly isolated to the 1020–1025 cm^−1^ region (C–O stretching, C–C stretching in xylans and pectins), suggesting that residual non-cellulosic carbohydrates were the main spectral differences between cultivars related to ethanol yields (Fig. 3, RHS) [20, 21].

Other spectral variations between pretreated residues derived from different cultivars (identified in PLS components 2–6) explained much less of the variance in ethanol yield. PLS 2 could explain a further 6.5 % of the variation in ethanol yields—primarily using variation in absorbance at cellulose-associated wavenumbers (1000, 1030, 1103, and 1160 cm^−1^). PLS 3 described a further 7.7 % of the variation in ethanol yields, attributed mostly to residual pectin, (1600 cm^−1^, COO^−^ asymmetric stretching) (Fig. 3, RHS). The remaining components (4–6) explained less than 7 % of the remaining variation combined, highlighting subtle differences in spectral regions previously identified by higher components (Fig. 3, RHS).

More PLS components were needed to explain the variation in ethanol yields (grams per kilogram untreated straw) when correlating them against spectra collected from the original straw (Fig. 3, LHS). The majority variation in ethanol yields between cultivars (52.4 %) can be explained by 15.8 % of the intraspecific spectral variation between the untreated straw (PLS 1). The PLS loadings for PLS 1 identified this spectral variation was found at XG- and pectin-related absorbances: 1020 cm^−1^ (C–O stretching, C–C stretching in XG and pectins), 875 cm^−1^ (C1–H bending in XG and cellulose), 1600 cm^−1^ (COO^−^ asymmetric stretching in pectins) and 1730/40 cm^−1^ (C=O stretching vibration of alkyl ester in pectins) [20, 21].

The variation in spectra at 1600 and 1730/40 cm^−1^ in PLS 1 are particularly interesting as they suggest that straw containing a greater abundance of methylesters obtain higher ethanol yields [21]. The abundance of methylesters implicates homogalacturonans as important cell wall components in determining saccharification efficiency in this species. Unlike most cereal crop residues, *B. napus* is a dicotyledonous plant—with pectin-rich, type I CWs similar to the model plant *Arabidopsis* [13]. Genetic manipulation in other species, including *Arabidopsis*, has independently revealed that saccharification yields produced from CW material is related to pectin-methyl esterification [29, 30]. It is therefore interesting to see that these changes may also influence genotypic variation in saccharification quality between *B. napus* cultivars after pilot-scale processing.

The loadings for the second PLS component (PLS 2), which explained a further 26.4 % of the variation in ethanol yields, can also show variation in pectin-associated peaks—the largest being at 1115 cm^−1^ (C–O, C–C stretching in pectin). Minor cellulose-associated peaks also contribute to PLS 2: 1415 cm^−1^ (C–O, C–C stretching in cellulose) and 1160 cm^−1^ (O–C–O asymmetric stretching of the glycosidic bond in cellulose) (Fig. 3, LHS).

The main spectral differences in lower PLS components, for example PLS 3, explaining a further 7.0 % of variation in ethanol yield, included 1034 cm^−1^ (glucan/glucomannan ring vibrations) and 1580 cm^−1^ (CW proteins) [18, 19]. PLS 4 explained a further 9.2 % of the variation in ethanol yields, primarily from the variation in absorbance at 1408 cm^−1^ (COO^−^ symmetric stretching in pectins) (Fig. 3, LHS).

Although not shown, similar conclusions could be drawn from the PLS analysis of FT-IR spectra in relation to glucose release after saccharification, which were almost identical to those associated with ethanol yield.

## Conclusions

Significant variation in Glc, ethanol and fermentation inhibitor yields were observed between cultivars—despite being grown, harvested and analysed under identical conditions. Genotypic differences in straw quality were not simply governed by Glc concentration in the original material but by the integrity of the non-cellulosic components. Arabinose- and galactose-rich polymers contained within the original straw were implicated as limiting saccharification yields between cultivars. PLS regression modelling revealed additional cultivar-specific properties, such as homogalacturonan abundance, which are likely to alter ethanol yields between cultivars. These observations are important to those wishing to breed agricultural residues as a feedstock for biorefining—highlighting key targets for improvement already present in cultivars of the same species.

## Methods

### Straw samples

Seventeen *B. napus* cultivars were grown under field conditions at KWS UK Ltd., Cambridge, UK (+52°, 8′, 32.40″, −1°, 6′, 19.66″), in a randomised order, in adjacent 3 × 12 m plots. The cultivars selected were a genetically diverse selection of *B. napus* genotypes, representative of the most common sub-groups—winter OSR (WR), spring OSR (SR), fodder rapes (FR) and swede (SW) [31]. The cultivars analysed in this study were as follows: Canard (FR), Canberra x Courage (WR), Darmor (WR), Erglu (SR), Hansen x Gaspard (WR), Judzae (SW), Lincrown x Express (WR), Madrical x Recital (WR), Major (WR), POH285 Bolko (WR), Quinta (WR), Ramses (WR), Sensation NZ (SW), Shannon x Winner (WR), Slapka Slapy (unspecified), Slovenska Krajova (WR) and York (SW).

All cultivars were harvested at maturity (8 Aug. 2012). Approximately 3kg OSR straw was collected upon ejection from a combine harvester which directly threshed and chipped the straw from a single cultivar into 2–3 cm pieces. The straw sample was taken from the centre of each 3m strip to prevent contamination from adjacent cultivars. The straw was then stored in woven polypropylene bags in a dry, unheated room before analysis.

### Cellulase and chemicals

The cellulase cocktail used in this study was Cellic® CTec2 (Novozymes, Denmark) with a stock cellulase activity of 180 FPU/mL determined following Ghose [32]. Unless otherwise stated, all chemicals used were analytical grade, purchased from Sigma-Aldrich, UK.

### Steam explosion of OSR straw

A sample of OSR straw (1 kg FW) from each cultivar was steam exploded into hot water (6.6 L) at a near-optimum pretreatment severity (210 °C, 10 min) using a Cambi™ Steam Explosion Pilot Plant [18]. After steam explosion, the heating chamber was then cleared twice by applying 2–3 bars of pressure to dislodge the majority of residual material. The pretreated biomass was filtered immediately through a 100μm nylon mesh bag in a low-speed centrifuge. The solid and liquid products were measured, and a representative sample of each fraction was taken for analysis. The steam explosion unit was extensively rinsed between each pretreatment to prevent cross contamination between cultivars.

### Analysis of steam explosion liquors

The concentration of fermentation inhibitors (organic acids and furfural derivatives) retained in each liquor (water-soluble fraction created after steam explosion) was quantified by HPLC after filtration (96-well filter plate, 0.2 μm). A Flexar® FX-10 UHPLC instrument (PerkinElmer, UK) equipped with a refractive index (RI) and photodiode array (PDA) detector was used, separating samples using an Aminex HPX-87H organic acid analysis column (Bio-Rad Laboratories Ltd., UK) (65 °C, mobile phase 5 mM H_2_SO_4_, flow rate 0.5 mL/min).

### Chemical composition of the untreated and pretreated solids

The matter content of the pretreated solid produced from each cultivar was established using an infrared drying balance (Mettler LP16, Mettler-Toledo, Belgium) drying duplicate samples (0.5 g) at 105 °C, to constant mass. A sample of each steam-exploded solid and untreated material was frozen in liquid nitrogen and freeze-milled into a fine powder to gain a homogenous sample for chemical analysis (3 min, SPEX 6700 freezer/mill, Spex Industries, NJ) and dried to constant mass (40 °C, overnight). Samples of the dried steam exploded residue and untreated material were then acid-hydrolysed (72 % H_2_SO_4_, 20 °C, 3 h followed by dilution to 1 M, 100 °C, 2.5 h). The sugar composition of the solid was established by converting the monomeric sugars released into their aditol acetate derivatives and quantifying their abundance by gas chromatography [33]. 2-Deoxy-Glc was used as an internal standard. Uronic acid content of the same materials were established colorimetrically after a milder hydrolysis regime (72 % H_2_SO_4_, 20 °C, 3 h followed by dilution to 1 M, 100 °C, 1 h) following [34].

### Fourier transform infrared (FT-IR) spectroscopy

FT-IR spectra were collected in the 800–4000 cm^−1^ region for each freeze-milled sample using a dynamic alignment FT-IR spectrophotometer (Bio-Rad FTS 175C, Bio-Rad Laboratories, Cambridge, USA), resolution 2 cm^−1^, 64 scans. The sample was trapped in a Golden Gate™ diamond attenuated total reflectance (ATR) accessory (Specac, Slough, UK) before collection. Triplicate spectra were taken for each material, truncated (800–1800 cm^−1^), baseline corrected (to 1800 cm^−1^) and area normalised before analysis.

### Determining saccharification yields for each cultivar

A 1g (DW equivalent) sample of each pretreated solid was suspended in 20mL sodium acetate/acetic acid buffer (5 % substrate, 0.1 M, pH 5, 0.01 % thiomersal) in 30mL screw-topped vials (Sterilin, UK), held in a shaker plate incubator (50 °C, 150 RPM). Cellic® CTec2 was added to the equilibrated solutions at a cellulase dose of 0.2 mL/g substrate (ca. 36 FPU/g). Digestions were conducted in triplicate and the amount of Glc quantified after 96 h of incubation. The amount of cellulase-derived Glc was also quantified and subtracted from the total.

### Quantification of sugars in biomass hydrolysates (reducing sugars and Glc)

The concentration of reducing sugars in undiluted biomass hydrolysates was estimated using a multiplexed DNS assay, optimised for this purpose [35]. The precise concentration of Glc released during hydrolysis was determined using GOPOD reagent as follows. A 100μL sample of each supernatant was heated in a sealed PCR plate to denature the cellulase (100 °C, 5 min), diluted to within a readable range (0–2 g/L). A 5μL sample of the diluted solutions was then dispersed in 195 μL of GOPOD reagent (Megazyme International Ltd., Ireland) in a microtitre plate. The amount of Glc in each hydrolysate was quantified after 20 min of incubation (50 °C) by comparing the absorbance of the products (510 nm) against a set of Glc calibration standards. Plates were covered during incubation to minimise evaporation.

### Simultaneous saccharification and fermentation (SSF) of pretreated straw

A sample of each pretreated substrate was suspended in 10 mL solution with a final concentration of 5 % substrate in nitrogen base (Formedium, Hunstanton, UK) and held in 20mL screw-topped glass vials. Both cellulases (36 FPU cellulase/g substrate) and a concentrated yeast inoculum were added to each vial and incubated for 96 h, 40 °C.

The yeast inoculum used was a robust thermo-tolerant yeast (*Saccharomyces cerevisiae*, strain NCYC 2826, National Collection of Yeast Cultures, Norwich, UK) grown from a slope culture, inoculating 1 L of yeast mould (YM) broth (3 d, 25 °C) before centrifuging, discarding the supernatant and partially reconstituting the yeast in nitrogen base. The final solutions contained 3.83 × 10^7^ viable cells/mL when inoculated—assayed using a NucleoCounter® YC-100™ (ChemoMetec, Denmark). SSFs were conducted as three independent replicates, and the ethanol released from a cellulase + yeast control was subtracted from each sample.

Ethanol concentrations were quantified using HPLC using a Series 200 LC instrument (PerkinElmer, UK) equipped with an Aminex HPX-87P carbohydrate analysis column (Bio-Rad Laboratories Ltd., Hemel Hempstead, UK). The mobile phase used was ultrapure water (0.6 mL/min) and concentration quantified using a refractive index (RI) detector, comparing absorbance to a set of ethanol standards.

### Statistical analysis

All descriptive statistics were calculated using Microsoft Excel and one-way ANOVAs conducted using GenStat v. 13 (VSN International, Ltd.). PLS regression (plsregress) was conducted in Matlab® (MathWorks, USA) [36].

## Abbreviations

Ara: arabinose
CW: cell wall
DW: dry weight
FR: fodder rapes
FT-IR: Fourier transform infrared
Fuc: fucose
FW: fresh weight
Gal: galactose
Glc: glucose
Man: mannose
OSR: oilseed rape
PLS: partial least squares
Rha: rhamnose
RSD: relative standard deviation
SR: spring OSR
SW: swede
UA: uronic acids
WR: winter OSR
XG: xyloglucan
Xyl: xylose
